# Epidermal Barrier Function and Skin Homeostasis in Skin with Permanent and Adhesive Tattoos: A Cross-Sectional Study

**DOI:** 10.3390/jcm10040888

**Published:** 2021-02-22

**Authors:** Jose-Pablo Serrano-Serra, Trinidad Montero-Vilchez, Agustin Buendia-Eisman, Salvador Arias-Santiago

**Affiliations:** 1 Dermatology Department, Faculty of Medicine, University of Granada, 18012 Granada, Spain or joserranoserra@correo.ugr.es (J.-P.S.-S.); abuendia@ugr.es (A.B.-E.); salvadorarias@ugr.es (S.A.-S.); 2 Dermatology Department, Hospital Universitario Virgen de las Nieves, 18012 Granada, Spain; 3 Instituto de Investigación Biosanitaria GRANADA, 18012 Granada, Spain

**Keywords:** adhesives, antioxidants, epidermis, petrolatum, skin barrier, tattooing

## Abstract

Tattoos are a current trend, but their impact on skin homeostasis and epidermal barrier function is not well known. So, the aims of this study are (1) to investigate epidermal barrier function and skin homeostasis in skin with permanent tattoos, adhesive temporary tattoos and non-tattooed skin, and (2) to analyze the effect of petrolatum on skin with permanent and adhesive tattoos. In total, 67 tattoos were enrolled (34 permanent tattoos and 33 adhesive tattoos). Temperature, transepidermal water loss (TEWL), stratum corneum hydration (SCH), erythema and total antioxidant capacity (TAC) were measured in skin with permanent tattoos, adhesive tattoos and non-tattooed skin before and after petrolatum application. The temperature was lower (30.47 °C vs. 31.01 °C; *p* = 0.001) on skin with permanent tattoos than non-tattooed skin, while SCH (48.24 Arbitrary Units (AU) vs. 44.15 AU; *p* = 0.008) was higher. Skin with adhesive tattoos showed lower temperature, SCH (21.19 AU vs. 41.31 AU; *p* < 0.001) and TAC (1.27 microcoulombs (uC) vs. 3.48 uC; *p* < 0.001), and higher TEWL (8.65 g/h/m^2^ vs. 6.99 g/h/m^2^; *p* = 0.003), than non-tattooed skin. After petrolatum application, the temperature decreased on skin with permanent tattoos, and TEWL and SCH decreased on skin with adhesive tattoos. Adhesive tattoos may affect skin barrier function, while permanent tattoos may have a lower impact. Tattooed and non-tattooed skin responds in different ways to moisturizers.

## 1. Introduction

Tattoos have been present in various cultures throughout history, mainly in relation to specific groups or linked to certain mystical or religious practices [1]. Nowadays, tattoos have spread to wider social circles and their popularity has increased notably. Recently, Kluger et al. observed that the prevalence of tattooed people ranged from 11.70% to 31.50% depending on the country. Tattoos were more frequent in women and young people [2]. Furthermore, the prevalence of tattoos in Europe is increasing. Since 2003, Borkenhagen et al. have observed an increase in the tattooed population in Germany [3]. Kluger et al. also showed higher rates of tattooed people in France from 2010 to 2017 [4,5]. 

The tattooing process consists of puncturing the skin with fine mechanized needles, at a speed of 50 to 3000 times per minute. Puncture depth may vary between 1 and 4 mm [6]. Approximately 1 mg/cm^2^ of pigment will remain on the skin [7]. Once ink particles enter the dermis, they are ingested by macrophages [8,9]. They may then disseminate systemically throughout the body, reaching the lymph nodes or the liver [10,11].

The dyes used in the tattooing process are predominantly insoluble or semi-soluble pigments, to which water and other compounds are added. Black pigments are derived from soot and are designed for industrial use. Some dyes contain a large number of organic pigments, which are used in association with metals to obtain the ink composition. Some pigments have not been toxicologically tested for use on humans [7]. 

The rate of side effects associated with tattoos is around 2%, the most frequent being infectious, allergic and granulomatous complications [12]. The potential carcinogenic effects of tattoos is controversial [13]. Moreover, tattoos can hinder the early diagnosis of pigmented skin lesions [14]. 

Sticker or adhesive tattoos are a very popular kind of tattoo, especially among children, who are the most vulnerable group to suffer from the secondary effects of chemicals [15]. The main ingredients of the ink are colorants, although detailed information about their composition is usually unavailable. Potentially harmful substances have been found in these products, such as aromatic amines, heavy metals and adhesive substances, which may cause several side effects, such as skin irritation [16]. 

There is little information regarding the effect of tattoos on skin barrier function and skin homeostasis. It has been noted that tattoos decrease the sweat rate and increase sweat sodium concentration [6]. Furthermore, it has been observed that skin capacitance is higher in tattooed skin compared to non-tattooed skin [17]. Due to the increase in the number of people with tattoos and the lack of awareness about the repercussions of tattooing, research on the effect of tattoos on the skin is increasingly relevant.

Differences in the skin barrier function parameters measured in tattooed and non-tattooed skin are expected because of the skin damage inflicted by tattoos. The objective of this study was to compare the parameters of cutaneous homeostasis and epidermal barrier function (temperature, transepidermal water loss (TEWL), stratum corneum hydration (SCH), erythema and total antioxidant capacity (TAC)) in skin with permanent tattoos and temporary adhesive tattoos, compared to non-tattooed skin. It was also evaluated if tattooed and non-tattooed skin responds in different ways to moisturizer application.

## 2. Materials and Methods

### 2.1. Design

(A) A cross-sectional study to assess skin homeostasis differences between non-tattooed skin, skin with permanent tattoos and skin with adhesive tattoos.

(B) Before and after study in patients with tattoos to assess changes in skin homeostasis after the application of a moisturizer (petrolatum).

### 2.2. Study Population

Participants were recruited from January 2020 to March 2020 at the Dermatology Department of the Hospital Universitario Virgen de las Nieves in Granada. Patients who had a tattoo were invited to participate. They were people affected by skin diseases not associated with their tattoos. People who accompanied patients and other voluntary participants were also recruited.

- Inclusion criteria: People over 18 years old, who had got their tattoo more than one month ago (in the case of permanent tattoos), without a skin reaction during the evaluation and who signed the informed consent form.
- Exclusion criteria: People who did not sign the informed consent form or who presented a skin disorder associated with the tattoo, or any cutaneous active (with an outbreak in the previous five years) inflammatory disease (psoriasis, atopic dermatitis).

### 2.3. Study Variables Measurement

Homeostasis parameters related to epidermal barrier function were measured. SCH (in arbitrary units, using Corneometer^®^ CM 825, Mirocaya, Bilbao, Spain), TEWL (in g·m^−2^h^−1^, using Tewameter^®^ TM 300, Mirocaya, Bilbao, Spain), erythema index (in arbitrary units, using Mexameter^®^ MX 18, Mirocaya, Bilbao, Spain) and skin temperature (in °C, using Skin-Thermometer ST 500, Mirocaya, Bilbao, Spain) were measured by a Multi Probe Adapter (MPA, Courage + Khazaka electronic GmbH, Mirocaya, Bilbao, Spain). Total antioxidant capacity (TAC) was measured using a conductive hydrogel placed on the skin, applying an electrochemical method, with the eBQC^®^ device (Bioquochem S.L. (BQCkit), Madrid, Spain), and expressed in microcoulombs. TAC is divided into two sections: fast antioxidants (Q1) have lower oxidation potential than slow antioxidants (Q2) [18]. Antioxidant capacity parameters were measured once and the other parameters were measured ten times, their average being used for analysis. Measurements were taken on a tattooed area and on non-tattooed area located on the symmetrically opposite side of the body. 

Adhesive tattoos were placed on the volar forearm and a wet sponge was used to push firmly against the skin, then held around 30 s. The skin was dried and finally, after 20 min wearing the tattoo, homeostasis parameters were measured.

To evaluate the moisturizer effect, the same amount of petrolatum was then applied on both measured areas (tattooed and non-tattooed skin) and after 30 min, measurements were taken again. Antioxidant capacity was also only measured before petrolatum application. 

Other variables: age, gender, phototype, past systemic or dermatological pathology, and medication, smoking and drinking habits were collected. Additionally, information was obtained about the tattoo, such as time of evolution, color, anatomical place, pathological reactions, subjective problems and cosmetic care.

### 2.4. Statistical Analysis

Descriptive statistics were used to present the characteristics of the sample. Continuous data were expressed as the mean and standard deviation. The absolute and relative frequency distributions were calculated for qualitative variable. The normality of the distribution for each parameter was checked with the Kolmogorov–Smirnov test.

To compare continuous data between tattooed skin and non-tattooed skin, the student’s *t*-test for paired samples or the Wilcoxon signed-rank test were used depending on the normality of the distribution. The same procedure was carried out to compare data from moisturized and non-moisturized skin. To compare data from skin with permanent tattoos skin and adhesive tattoos, the student’s *t*-test for independent samples or the Mann–Whitney U test were used depending on the normality of the distribution. The Pearson correlation coefficient was calculated to test for possible correlation between continuous variables. Two-sided *p*-values < 0.05 were considered as the level of statistical significance. SPSS version 24.0 (SPSS Inc, Chicago, IL, USA) was used for statistical analyzes.

## 3. Results

### 3.1. Participants’ Features

This study included 67 samples (34 permanent and 33 adhesive tattoos) and 67 controls. The baseline characteristics of participants are given in Table 1.

Regarding permanent tattoos, 82.40% used black pigments only (28/34). Men had 52.90% of the tattoos (18/34). The mean age of participants in this group was 26.12 (SD 4.74) years old. The mean age of the tattoos was 24.62 (SD 24.33) months. The most frequent anatomical site for a tattoo was the upper limbs (19/34). Three past adverse events associated with tattoos were reported: one allergic reaction, and two itchiness associations.

Regarding adhesive tattoos, 54.50% were on males (18/33) and the mean age of the participants was 26.45 (SD 9.82) years old. All the adhesive tattoos were on the arm and had more than one color. 

### 3.2. Skin Homeostasis Analysis between Skin with Permanent Tattoos and Non-Tattooed Skin

The skin homeostasis parameters in skin with permanent tattoos and non-tattooed skin are summarized in Table 2. 

Temperature was lower in tattooed skin than in non-tattooed skin (30.47 °C vs. 31.01 °C; *p* = 0.001), while SCH (48.24 AU vs. 44.15 AU; *p* = 0.008) was higher in tattooed skin. The TEWL and TAC values did not differ between tattooed and non-tattooed skin before petrolatum application.

After petrolatum application, the temperature of tattooed skin was lower (30.18 °C vs. 30.61 °C; *p* < 0.001) than non-tattooed skin, while SCH was higher in tattooed skin (44.32 AU vs. 40.78 AU; *p* = 0.047). No differences were found in TEWL values after moisturization.

### 3.3. Skin Homeostasis Analysis between Skin with Adhesive Tattoos and Non-Tattooed Skin

Skin homeostasis changes were found between skin with adhesive tattoos and non-tattooed skin (Table 3). 

Statistically significant differences were found in non-moisturized samples. Temperature (30.78 °C vs. 32.05 °C; *p* < 0.001), SCH (21.19 AU vs. 41.31 AU; *p* < 0.001) and TAC (1.27 microcoulombs (uC) vs. 3.48 uC; *p* < 0.001) were lower in skin with adhesive tattoos skin, while TEWL (8.65 g/h/m^2^ vs. 6.99 g/h/m^2^; *p* = 0.003) was higher in skin with adhesive tattoos. 

Similar results were obtained after the skin was moisturized, except for non-significant differences in TEWL.

### 3.4. Skin Homeostasis Analysis between Skin with Permanent Tattoos and Skin with Adhesive Tattoos

Differences were found between skin with permanent tattoos and skin with adhesive tattoos (Table 4). 

Skin with permanent tattoos presented higher SCH (48.24 AU vs. 21.19 AU; *p* < 0.001) and TAC (5.40 uC vs. 1.27 uC; *p* < 0.001) values before the petrolatum application. Similar TEWL values were observed in skin with permanent and adhesive tattoos.

### 3.5. Skin Homeostasis Analysis Comparing before and after Petrolatum Application

Changes were observed between non-tattooed skin, skin with permanent tattoos and skin with adhesive tattoos after petrolatum application (Table 5). 

The temperature decreased by −0.36 (SD 0.73) °C in non-tattooed skin (*p* = 0.008) and −0.26 (SD 0.74) °C in skin with permanent tattoos (*p* = 0.050) after petrolatum application. No differences in SCH and TEWL were observed after applying petrolatum to skin with permanent tattoos or non-tattooed skin.

After applying petrolatum to skin with adhesive tattoos, the temperature increased by 1.05 °C (SD 0.89) (*p* < 0.001), TEWL decreased by −1.35 (SD 3.28) g/h/m^2^ (*p* = 0.025) and SCH decreased by −4.83 (SD 5.00) AU (*p* < 0.001).

The effects of petrolatum on erythema were different in non-tattooed skin and tattooed skin. Erythema decreased by −21.56 (SD 5.25) AU in non-tattooed skin (*p* < 0.001), while no effect was observed in skin with permanent tattoos (*p* = 0.875) or skin with adhesive tattoos (*p* = 0.396). 

## 4. Discussion

Permanent tattoos do not significantly affect skin homeostasis compared to non-tattooed skin. Only temperature and erythema decreases were observed. Temporary adhesive tattoos alter cutaneous homeostasis, as they reduce temperature, SCH and antioxidant capacity, and increase TEWL, compared to non-tattooed skin. Petrolatum reduces TEWL with adhesive tattoos.

To the best of our knowledge, only one previous study has compared skin homeostasis parameters between permanent tattoos and non-tattooed skin [17]. Norrelet et al. found no differences in TEWL and pH values between non-tattooed and tattooed skin in 28 tattoos. Similarly, they found higher capacitance on tattooed skin [17], in agreement with the higher SCH values on tattooed skin observed in our study. Skin barrier disruption is observed after needle injury micro-traumatisms, which is shown by the slight increases in TEWL observed in our report. As it has been previously shown, this increased water flow may indirectly lead to increased hydration [19]. 

There are no studies regarding the epidermal barrier function of skin with adhesive tattoos. Increased TEWL values are found after the application and removal of adhesives, such as wound dressing, resulting in skin barrier impairment [20]. Our study showed higher TEWL in skin with adhesive tattoos, associated with skin damage. SCH and TAC were also altered in skin with adhesive tattoos. Since high TEWL and low SCH values indicate damage to the skin barrier function [21], and adhesive modifies these parameters, adhesive tattoos may negatively affect the skin. In that way, people suffering from cutaneous disease with a skin barrier dysfunction, such as psoriasis or atopic dermatitis [22,23], should avoid repeated use of adhesive tattoos. Moreover, these findings should be considered in the development of new electronic adhesive devices for medical use, which could also damage the skin [24]. Future studies on this matter may determine if skin properties are affected by the use of adhesive medical devices. 

Skin with adhesive tattoos showed lower antioxidant values compared to non-tattooed skin. Previously, it has also been observed that tattoo exposure induces mild oxidative stress and enhances the activity of certain antioxidants [25]. The composition of adhesive tattoos is usually unknown, but they usually contain colorants and hazardous chemicals, such as phthalate ester, aromatic amines, heavy metals or even adhesive substances [16], which may lead to skin irritation, releasing proinflammatory cytokines and inducing oxidative stress [26], which may explain the decreased antioxidant capacity of adhesive tattooed skin.

Petrolatum was used to assess if tattooed skin reacts similarly to non-tattooed skin after moisturizing. Petrolatum is known to reduce TEWL on healthy skin [27]. Our study did not show significant differences for TEWL in moisturized and non-moisturized skin after a single application, which is consistent with previous reports [28]. The petrolatum effect in improving TEWL might be observed for long-term application, which involves creating a water-repellent lipid layer on the skin surface that blocks water loss [29]. However, a significant decrease in TEWL after a single petrolatum application was observed in adhesive tattooed skin. Petrolatum has been proven to help to reduce erythema following skin damage induced by different agents, such as ultraviolet radiation or topical medication [30,31]. Petrolatum is a hypoallergenic substance that does not bind to proteins or undergo chemical alteration in the skin [32].

This study was subject to several limitations: a limited sample size and a short follow-up period. Future research could measure the skin barrier function parameters of other types of temporary tattoos, such as henna or jagua, which have been reported to cause skin pathologies, especially allergic reactions [33,34].

## 5. Conclusions

Permanent tattoos do not significantly affect skin barrier function. Adhesive tattoos cause skin barrier dysfunction, showing increased TEWL and decreased SCH values, as well as increased temperature and decreased antioxidant skin capacity. Tattooed and non-tattooed skin respond in different ways to moisturizers.

## Figures and Tables

**Table 1:** Descriptive characteristics of permanent and adhesive tattoos and the subjects they belong to.

Sociodemographic Features	Permanent Tattoos (*n* = 34)	Adhesive Tattoos (*n* = 33)
Age (years) (mean (range))	26.12 (21–41)	26.45 (19–53)
Gender, *n* (%)		
Male	18 (52.9%)	18 (54.5%)
Female	16 (47.1%)	15 (45.5%)
Phototype, *n* (%)		
I	1 (2.9%)	0 (0%)
II	4 (11.8%)	4 (12.1%)
III	23 (67.6%)	25 (75.8%)
IV	6 (17.6%)	4 (12.1%)
History of cutaneous pathology, *n* (%)		
Neoplasm	2 (5.9%)	0 (0%)
Atopic dermatitis	6 (17.6%)	6 (18.2%)
Other dermatitis	2 (5.9%)	0 (0%)
Systemic medication, *n* (%)		
Contraceptives	9 (26.5%)	8 (24.2%)
Other	3 (8.8%)	3 (9.1%)
Topic medication, *n* (%)		
Corticoids	5 (14.7%)	2 (6.1%)
Other	0 (0%)	2 (6.1%)
Smokers		
*n* (%)	9 (26.5%)	2 (6.1%)
Mean smoking pack per day (range)	1.31 (0–25)	0.24 (0–4)
Drinkers, *n* (%)		
*n* (%)	19 (55.9%)	18 (54.5%)
Mean alcohol unit/day (range)	0.27 (0–1.14)	0.25 (0–1)
Tattoo duration (months) (mean (range))	24.62 (1–108)	-
Pathologic reaction, *n* (%)		
Cutaneous allergy	1 (2.9%)	0 (0%)
Itchiness	2 (5.9%)	0 (0%)
Regular moisturizing, *n* (%)	8 (23.5%)	0 (0%)
Localization, *n* (%)		
Head and neck	3 (8.8%)	0 (0%)
Upper limbs	19 (55.9%)	33 (100%)
Trunk	8 (23.5%)	0 (0%)
Lower limbs	4 (11.8%)	0 (0%)
Color, *n* (%)		
Black pigments only	28 (82.4%)	0 (0%)
Other combinations	6 (17.6%)	33 (100%)

Data are expressed as relative (absolute) frequencies and means (standard deviation (SD).

**Table 2:** Differences in biophysical parameters between skin with permanent tattoos and non-tattooed skin.

	Before Moisturizer (*n* = 34)			After Moisturizer (*n* = 34)		
	Skin with Permanent Tattoos	Non-Tattooed Skin	*p*	Skin with Permanent Tattoos	Non-Tattooed Skin	*p*
Temperature (°C)	30.47 (1.46 SD)	31.01 (1.27 SD)	0.001 *	30.18 (1.37 SD)	30.61 (1.32 SD)	<0.001 **
TEWL (g/h/m^2^)	8.72 (5.42 SD)	8.36 (5.80 SD)	0.857	7.90 (4.03 SD)	8.84 (5.67 SD)	0.433
SCH (AU)	48.24 (16.95 SD)	44.15 (15.26 SD)	0.008 *	44.32 (13.05 SD)	40.78 (13.17 SD)	0.047 **
Erythema (AU)	96.77 (141.43 SD)	280.08 (88.76 SD)	<0.001 *	98.33 (142.78 SD)	258.00 (94.46 SD)	<0.001 **
Q1 (uC)	0.97 (0.46 SD)	1.08 (0.64 SD)	0.490	-	-	-
Q2 (uC)	4.44 (1.36 SD)	4.13 (1.21 SD)	0.235	-	-	-
QT (uC)	5.40 (1.71 SD)	5.20 (1.50 SD)	0.598	-	-	-

AU, arbitrary units; SCH, stratum corneum hydration; TEWL, transepidermal water loss; uC, microcoulombs; Q1, fast antioxidant capacity; Q2, slow antioxidant capacity; QT, total antioxidant capacity. The *p* value after using Student *t*-test for paired samples or Wilcoxon signed-rank test where necessary, depending on the normality of the distribution, to compare biophysical parameters between skin with permanent tattoos and non-tattooed skin. * *p* value after using Student’s *t* test for paired samples to compare skin with permanent tattoos and non-tattooed skin before moisturizer. ** *p* value after using Student’s *t* test for paired samples to compare skin with permanent tattoos and non-tattooed skin after moisturizer.

**Table 3:** Differences in biophysical parameters between skin with adhesive tattoos and non-tattooed skin.

	Before Moisturizer (*n* = 33)			After Moisturizer (*n* = 33)		
	Skin with Adhesive Tattoos	Non-Tattooed Skin	*p*	Skin with Adhesive Tattoos	Non-Tattooed Skin	*p*
Temperature (°C)	30.78 (1.11 SD)	32.05 (0.86 SD)	<0.001 *	31.83 (0.68 SD)	32.32 (0.66 SD)	<0.001 **
TEWL (g/h/m^2^)	8.65 (3.11 SD)	6.99 (2.10 SD)	0.003 *	7.31 (2.14 SD)	7.88 (1.63 SD)	0.062
SCH (AU)	21.19 (6.71 SD)	41.31 (9.18 SD)	<0.001 *	16.37 (7.27 SD)	31.45 (9.99 SD)	<0.001 **
Erythema (AU)	312.20 (211.14 SD)	212.61 (37.01 SD)	0.021 *	335.34 (225.93 SD)	194.67 (38.88 SD)	0.001 **
Q1 (uC)	0.17 (0.17 SD)	0.62 (0.38 SD)	<0.001 *	-	-	-
Q2 (uC)	1.10 (0.76 SD)	2.86 (1.18 SD)	<0.001 *	-	-	-
QT (uC)	1.27 (0.91 SD)	3.48 (1.51 SD)	<0.001 *	-	-	-

AU, arbitrary units; SCH, stratum corneum hydration; TEWL, transepidermal water loss; uC, microcoulombs; Q1, fast antioxidant capacity; Q2, slow antioxidant capacity; QT, total antioxidant capacity. The *p* value after using Student *t*-test for paired samples or Wilcoxon signed-rank test where necessary, depending on the normality of the distribution, to compare biophysical parameters between skin with adhesive tattoos and non-tattooed skin. * *p* value after using Student’s *t* test for paired samples to compare skin with adhesives tattoos and non-tattooed skin before moisturizer. ** *p* value after using Student’s *t* test for paired samples to compare skin with adhesives tattoos and non-tattooed skin after moisturizer.

**Table 4:** Differences in biophysical parameters between skin with permanent tattoos and skin with adhesive tattoos.

	Before Moisturizer			After Moisturizer		
	Skin with Permanent Tattoos (*n* = 34)	Skin with Adhesive Tattoos (*n* = 33)	*p*	Skin with Permanent Tattoos (*n* = 34)	Skin with Adhesive Tattoos (*n* = 33)	*p*
Temperature (°C)	30.47 (1.46 SD)	30.78 (1.11 SD)	0.322	30.18 (1.37 SD)	31.83 (0.68 SD)	<0.001 **
TEWL (g/h/m^2^)	8.72 (5.42 SD)	8.65 (3.11 SD)	0.132	7.90 (4.03 SD)	7.31 (2.14 SD)	0.888
SCH (AU)	48.24 (16.95 SD)	21.19 (6.71 SD)	<0.001 *	44.32 (13.05 SD)	16.37 (7.27 SD)	<0.001 **
Erythema (AU)	96.77 (141.43 SD)	312.20 (211.14 SD)	<0.001 *	98.33 (142.78 SD)	335.34 (225.93 SD)	<0.001 **
Q1 (uC)	0.97 (0.46 SD)	0.17 (0.17 SD)	<0.001 *	-	-	-
Q2 (uC)	4.44 (1.36 SD)	1.10 (0.76 SD)	<0.001 *	-	-	-
QT (uC)	5.40 (1.71 SD)	1.27 (0.91 SD)	<0.001 *	-	-	-

AU, arbitrary units; SCH, stratum corneum hydration; TEWL, transepidermal water loss; uC, microcoulombs; Q1, fast antioxidant capacity; Q2, slow antioxidant capacity; QT, total antioxidant capacity. The *p* value after using Student’s *t*-test for independent samples or Mann–Whitney U test where necessary, depending on the normality of the distribution, to compare biophysical parameters between permanent tattooed skin and adhesive tattooed skin. * *p* value after using Student’s *t* test for independent samples to compare skin with permanent tattoos and skin with adhesive tattoos before moisturizer. ** *p* value after using Student’s *t* test for independent samples to compare skin with permanent tattoos and skin with adhesive tattoos after moisturizer.

**Table 5:** Differences in biophysical parameters between moisturized skin and non-moisturized skin for non-tattooed skin, skin with permanent tattoos and skin with adhesive tattoos.

	Non-Tattooed Skin (*n* = 34)			Skin with Permanent Tattoos (*n* = 34)			Skin with Adhesive Tattoos (*n* = 33)		
	After Moisturization	Mean Difference between before and after Moisturization	*p* Value Comparing before vs. after Moisturization	After Moisturization	Mean Difference between before and after Moisturization	*p* Value Comparing before vs. after Moisturization	After Moisturization	Mean Difference between before and after Moisturization	*p* Value Comparing before vs. after Moisturization
Temperature (°C)	30.61 (1.32 SD)	−0.36 (0.73 SD)	0.008 *	30.18 (1.37 SD)	−0.26 (0.74 SD)	0.050 **	31.83 (0.68 SD)	+1.05 (0.89 SD)	<0.001 ***
TEWL (g/h/m^2^)	8.84 (5.67 SD)	+0.33 (6.88 SD)	0.268	7.90 (4.03 SD)	−0.83 (6.32 SD)	0.903	7.31 (2.14 SD)	−1.35 (3.28 SD)	0.025 ***
SCH (AU)	40.78 (13.17 SD)	−4.01 (17.49 SD)	0.197	44.32 (13.05 SD)	−4.26 (18.15 SD)	0.187	16.37 (7.27 SD)	−4.83 (5.00 SD)	<0.001 ***
Erythema (AU)	258.85 (94.46 SD)	−21.56 (30.16 SD)	<0.001 *	98.33 (142.78 SD)	+2.29 (24.76 SD)	0.875	335.34 (225.93 SD)	+23.14 (143.93 SD)	0.396

AU, arbitrary units; SCH, stratum corneum hydration; TEWL, transepidermal water loss. The *p* value after using Student’s *t*-test for paired samples or Wilcoxon signed-rank test where necessary, depending on the normality of the distribution, to compare biophysical parameters between moisturized skin and non-moisturized skin in skin with permanent tattoos, adhesive tattoos and non-tattooed skin. * *p* value after using Student’s *t* test for paired samples to compare skin homeostasis parameters on non-tattooed skin before and after moisturization. ** *p* value after using Student’s *t* test for paired samples to compare skin homeostasis parameters on skin with permanent tattoos before and after moisturization. *** *p* value after using Student’s *t* test for paired samples to compare skin homeostasis parameters on skin with adhesive tattoos before and after moisturization.

## Data Availability

The data presented in this study are available on request from the corresponding author.
